# Multidisciplinary Management of Severe Iatrogenic Injury to the Maxillary Incisors Following Mesiodens Extraction

**DOI:** 10.7759/cureus.110289

**Published:** 2026-06-05

**Authors:** Prabhleen Brar, Sudhir Bhandari, Raj K Verma

**Affiliations:** 1 Department of Conservative Dentistry and Endodontics, Postgraduate Institute of Medical Education and Research, Chandigarh, IND; 2 Department of Prosthodontics, Postgraduate Institute of Medical Education and Research, Chandigarh, IND; 3 Department of Orthodontics, Postgraduate Institute of Medical Education and Research, Chandigarh, IND

**Keywords:** cone-beam computed tomography, iatrogenic disease, mesiodens, multidisciplinary care, tooth injuries

## Abstract

Iatrogenic injury during mesiodens extraction may result in severe damage to adjacent teeth and supporting structures, particularly when adequate imaging and surgical planning are lacking. This case report describes the multidisciplinary management of extensive iatrogenic trauma involving the maxillary anterior teeth following surgical removal of a mesiodens. A 28-year-old woman presented with grade II mobility of the maxillary incisors (#12, #11, #21, and #22) six months after mesiodens extraction. Clinical and cone-beam computed tomography (CBCT) examinations revealed extensive root destruction and loss of supporting alveolar bone involving the teeth. A conservative tooth-preserving treatment approach was adopted, including root canal therapy with mineral trioxide aggregate obturation, surgical apicectomy, fiber-reinforced composite splinting, and definitive zirconia prosthetic rehabilitation. Clinical and radiographic follow-up at 12 months demonstrated satisfactory healing, reduction in mobility, and stable functional and esthetic outcomes. This case emphasizes the importance of CBCT-guided surgical planning and interdisciplinary management in preventing and successfully treating complex iatrogenic dental injuries.

## Introduction

Iatrogenic injury is defined as unintended damage to tissues or anatomical structures resulting from medical or dental intervention [1]. In dentistry, such injuries may arise because of inadequate diagnosis, improper treatment planning, insufficient surgical expertise, or failure to recognize the anatomical relationship between adjacent structures [2]. Among the various developmental anomalies encountered in the anterior maxilla, mesiodens is the most common supernumerary tooth, typically located between the maxillary central incisors. Although mesiodens extraction is considered a routine minor oral surgical procedure, its close proximity to adjacent teeth, periodontal tissues, and developing roots poses a significant risk of complications when performed without precise localization and careful surgical execution [3]. Potential complications include root damage, luxation, devitalization of adjacent teeth, periodontal defects, loss of supporting bone, and soft tissue trauma, all of which may compromise both esthetics and function in the anterior region [4].

With advancements in diagnostic imaging, cone-beam computed tomography (CBCT) has emerged as an invaluable tool for the management of impacted and ectopic supernumerary teeth. CBCT provides accurate three-dimensional visualization of the tooth position and its relationship to adjacent anatomical structures, thereby facilitating minimally invasive surgical approaches and reducing the risk of intraoperative and postoperative complications [5]. Despite these advancements, the inadequate utilization of advanced imaging modalities and improper surgical judgment may lead to severe iatrogenic trauma. Such complications can create complex clinical situations that require multidisciplinary rehabilitation involving endodontic, periodontal, restorative, and surgical management [6]. Preservation of the affected teeth and surrounding tissues becomes particularly challenging when extensive hard- and soft-tissue destruction occurs in the esthetically sensitive maxillary anterior region [7].

The present case report describes successful multidisciplinary management of severe iatrogenic injury to the maxillary anterior teeth following mesiodens extraction. This case highlights the consequences of inadequate surgical management and emphasizes the importance of careful preoperative assessment, appropriate imaging, and meticulous surgical planning. Through coordinated interdisciplinary treatment, functional and esthetic rehabilitation of the affected teeth and supporting tissues was achieved. Clinical and radiographic evaluations at the two-year follow-up demonstrated favorable healing, periodontal stability, and satisfactory functional outcomes.

## Case presentation

A 28-year-old woman with a non-contributory medical history presented to the Department of Dentistry at the Post Graduate Institute of Medical Education and Research (PGIMER), Chandigarh, with a chief complaint of mobility in the upper anterior teeth for approximately six months. The patient reported no associated pain, swelling, or discomfort during mastication. Her dental history revealed that she had undergone surgical extraction of an impacted mesiodens at a private dental clinic six months earlier. Following the procedure, the treating dentist informed the patient of the possibility of damage and resorption of the adjacent maxillary incisors and advised extraction or root canal treatment. The patient was referred to our institution for a second opinion and a tooth-preserving solution.

An extraoral examination revealed no facial asymmetry or swelling. Intraoral examination revealed grade II mobility in teeth #12, #11, #21, and #22. The gingival tissues appeared healthy with no sinus tract formation or periodontal pockets exceeding 3 mm. The teeth were nontender to percussion and palpation. Thermal and electric pulp sensibility testing (Digitest 3 Pulp Tester, Parkell Inc., Brentwood, New York, USA) elicited delayed or absent responses in the involved incisors.

Intraoral periapical radiography demonstrated irregular periapical radiolucencies and discontinuity of root morphology involving the maxillary incisors (Figure 1).

**Figure 1:**
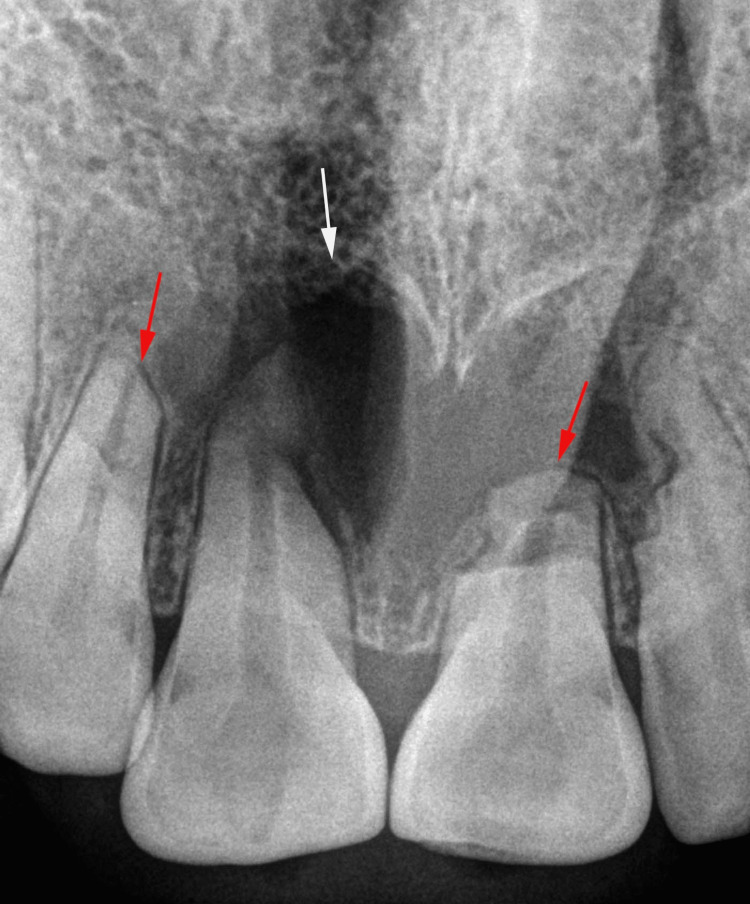
Intraoral periapical radiograph demonstrating a radiolucent lesion with diffuse borders (white arrow) and root resorption of the incisors (red arrows)

CBCT (Carestream CS 9300, Carestream Dental, Atlanta, USA) revealed extensive destruction of the apical and middle thirds of the roots of teeth #12, #11, #21, and #22, associated with a large (3 cm × 2 cm) anterior osseous defect extending across the premaxillary region (Figure 2). The defect exhibited a well-defined radiolucent appearance with corticated borders, representing a vertical/angular bone defect with complete loss of labial and palatal cortical plates. A significant loss of supporting alveolar bone and external root structure was evident, contributing to the observed mobility.

**Figure 2:**
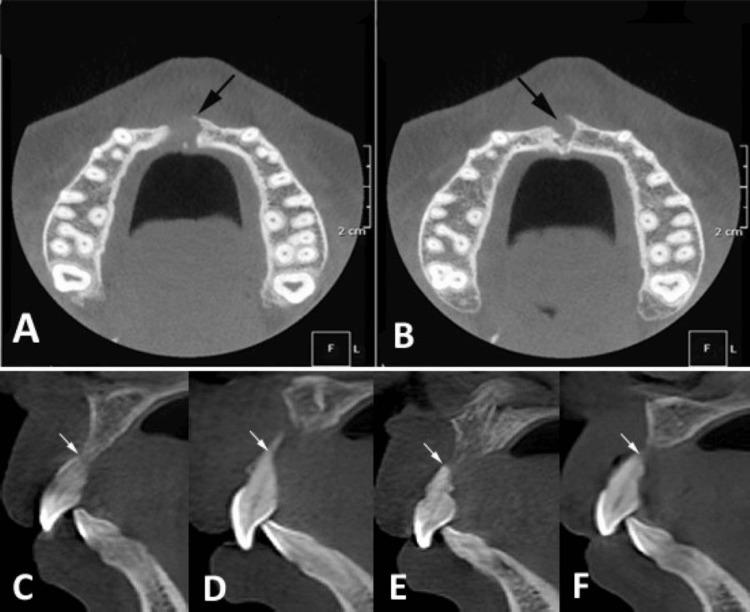
CBCT images demonstrating (A) radiolucency in the anterior maxillary region (black arrow) in the axial view; (B) destruction of the labial cortical plate (black arrow) in the anterior maxilla in the axial view; and (C-F) external root resorption (white arrow) involving teeth #12 (C), #11 (D), #21 (E), and #22 (F) in the labiopalatal view CBCT: cone-beam computed tomography

The patient provided preoperative CBCT records obtained prior to mesiodens extraction (Figure 3). Evaluation of these images demonstrated intact root morphology and normal surrounding alveolar bone around all the maxillary incisors. The scan revealed horizontally impacted mesiodens positioned palatally between the roots of the maxillary central incisors. The cusp tip of the mesiodens was located approximately 13.2 mm above the occlusal plane. The supernumerary tooth exhibited a conical crown form with a single fully formed root and canal. Interestingly, both internal and external crown resorption were evident within the mesiodens. The proximity of the mesiodens to the adjacent incisor roots suggested a high-risk surgical relationship.

**Figure 3:**
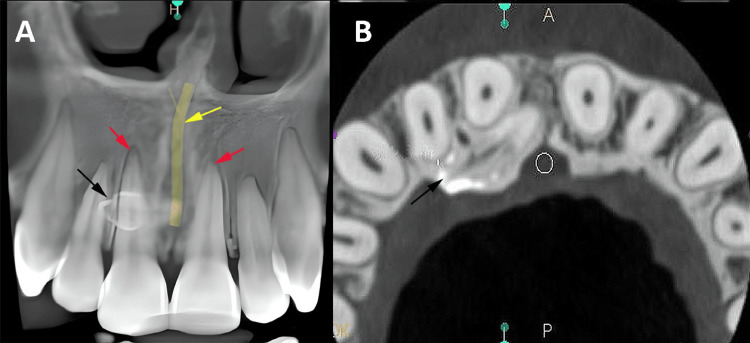
Preoperative CBCT images showing (A) an impacted mesiodens (black arrow), the nasopalatine canal (yellow arrow), and mature root apices of the incisor teeth (red arrows); and (B) the impacted mesiodens (black arrow) in the axial view CBCT: cone-beam computed tomography

Comparative evaluation of pre- and post-extraction CBCT scans confirmed extensive iatrogenic damage to the adjacent permanent incisors during the surgical removal of the mesiodens. The injury resulted in irreversible loss of root hard tissue, secondary inflammatory bone resorption, and compromised periodontal support, ultimately leading to the pathological mobility of the involved teeth.

Treatment

After a detailed discussion with the patient, three treatment options were proposed: extraction of the affected incisors followed by bone grafting and implant-supported rehabilitation; extraction followed by a removable partial denture prosthesis; or a multidisciplinary tooth-preserving approach involving endodontic therapy, surgical management, splinting, and definitive prosthodontic rehabilitation.

Considering the patient’s young age and desire to preserve her natural dentition, a third treatment option was chosen. Conventional root canal treatment was initiated for teeth #12, #11, #21, and #22, under rubber dam isolation. Cleaning and shaping were performed using rotary nickel-titanium instruments (Protaper Gold, Dentsply Sirona, Ballaigues, Switzerland) with copious irrigation using sodium hypochlorite (Prime Dental Products Pvt. Ltd., Mumbai, India) and saline. Owing to extensive apical root destruction and the need for enhanced sealing ability, mineral trioxide aggregate (MTA) (MTA Angelus, Angelus Dental Solutions, Londrina, Brazil) was used as the obturating material in all the canals.

Following the completion of endodontic therapy, surgical apicectomy was performed under local anesthesia. A full-thickness mucoperiosteal flap was reflected in the anterior maxilla, and necrotic, structurally compromised root ends were resected. Root-end preparation and sealing were achieved using MTA to establish an adequate apical barrier and improve the long-term prognosis. Thorough debridement of granulation tissue was performed before flap repositioning and suturing.

To stabilize the mobile teeth and facilitate periodontal healing, a physiological fiber-reinforced composite splint (Ribbond®, Ribbond Inc., Seattle, Washington, USA) extending from the canine to the canine was placed. The patient was instructed on meticulous oral hygiene maintenance and scheduled for periodic clinical and radiographic follow-up (Figure 4).

**Figure 4:**
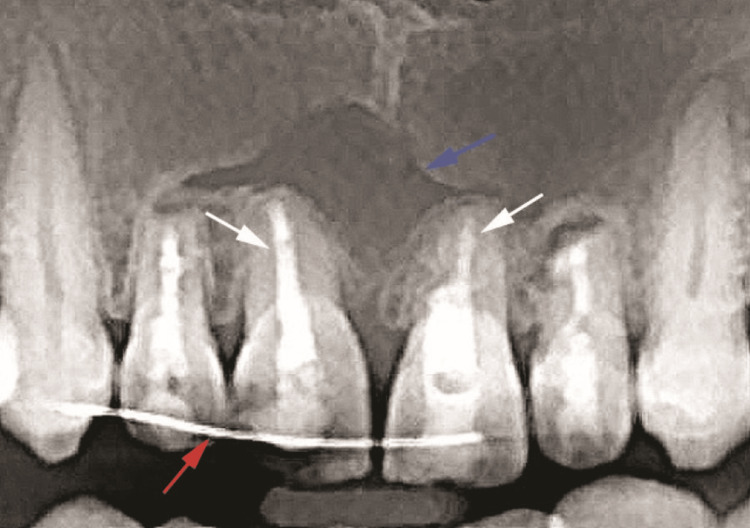
Panoramic CBCT view after six months demonstrating MTA obturation of teeth #12, #11, #21, and #22 (white arrows), a healed radiolucent lesion in the anterior maxillary region (blue arrow), and stabilization with a fiber splint extending across the anterior teeth (red arrow) CBCT: cone-beam computed tomography, MTA: mineral trioxide aggregate

After 12 months of successful stabilization and healing, definitive prosthodontic rehabilitation was completed with multilayer zirconia fixed dental prostheses (Katana Zirconia, Kuraray Noritake Dental, Tokyo, Japan) (Figure 5). Teeth #12 and #11 were restored as a single splinted unit, whereas #21 and #22 were restored as a second splinted unit to optimize esthetics, function, and load distribution.

**Figure 5:**
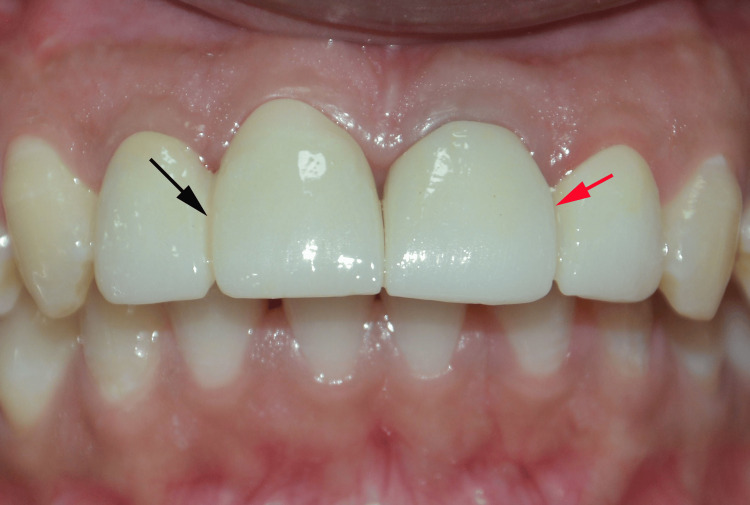
Twelve-month postoperative follow-up showing splinted units #11 and #12 (black arrow) and #21 and #22 (red arrow), all rehabilitated with zirconia crowns

Outcome and follow-up

The patient was periodically reviewed at 3, 6, and 12 months. Clinical and radiographic evaluations were performed at each follow-up visit. A progressive reduction in mobility and satisfactory soft tissue healing were observed throughout the follow-up period.

At the 12-month follow-up, the patient remained asymptomatic and expressed satisfaction with both the esthetic and functional outcomes. Clinical examination revealed an absence of tenderness and complete resolution of pathological mobility. Periapical radiographs showed satisfactory healing, stable bone architecture, and no evidence of progressive external root resorption or periapical pathology. The splinted zirconia prostheses remained functional and esthetically acceptable, indicating successful preservation of the affected dentition through a multidisciplinary management approach.

## Discussion

Mesiodens extraction is generally considered a routine surgical procedure; however, its close anatomical relationship with adjacent permanent incisors may predispose to serious complications when performed without adequate planning and surgical precision [8]. The present case demonstrated extensive iatrogenic damage to the maxillary anterior teeth following mesiodens removal, resulting in severe root destruction, loss of supporting bone, and pathological mobility. Such complications emphasize the importance of a comprehensive preoperative assessment and careful surgical execution during the management of impacted supernumerary teeth. Similar severe sequelae following mesiodens extraction were reported by Machado and Braga, who described extensive trauma to adjacent incisors associated with improper surgical manipulation, ultimately compromising tooth prognosis [4].

Accurate localization of impacted mesiodens is essential for minimizing surgical complications. Conventional two-dimensional radiographs often fail to provide precise spatial relationships between mesiodens and adjacent structures [9]. In the present case, retrospective evaluation of preoperative CBCT scans clearly demonstrated the intimate proximity of the impacted mesiodens to the roots of the maxillary incisors, indicating a high-risk surgical situation. A previous study highlighted the significance of CBCT-based surgical planning in improving the safety and predictability of supernumerary tooth removal by enabling three-dimensional visualization and reducing intraoperative trauma [5]. Likewise, Rodrigues et al. emphasized that inadequate radiographic assessment is a major contributor to dental iatrogenesis and treatment failure [6]. These findings strongly support the importance of advanced imaging in preventing avoidable complications during oral surgeries.

The extensive root damage and compromised periodontal support observed in this case necessitated a multidisciplinary treatment approach to preserve the natural dentition. Despite the poor initial prognosis, tooth preservation was attempted because of the patient’s young age, esthetic concerns, and desire to avoid extraction and implant rehabilitation. Similar conservative rehabilitation strategies for traumatized anterior teeth have been advocated in a study, which reported successful preservation of severely compromised maxillary incisors through coordinated endodontic and restorative management [1]. This case further reinforces the concept that even structurally compromised teeth may remain functional when appropriate interdisciplinary treatment is implemented.

MTA was selected as the obturation and retrograde filling material because of its superior sealing ability, biocompatibility, and capacity to promote hard tissue healing. Previous studies have demonstrated favorable outcomes with MTA in cases of apical root defects, perforations, and surgical endodontic management [10]. The successful clinical and radiographic healing observed in the present case supports the established regenerative potential of MTA in managing teeth with severe apical destruction [11]. Surgical debridement and apicectomy further contribute to the elimination of inflamed tissues and establishment of a favorable environment for periapical healing.

Stabilization of the affected teeth using a fiber-reinforced composite splint plays a critical role in reducing mobility and facilitating periodontal healing. Splinting helps to distribute occlusal forces and provides functional stability during the healing phase, particularly in teeth with compromised periodontal support [12]. Long-term stabilization, combined with definitive prosthodontic rehabilitation using splinted zirconia restorations, helps maintain esthetics and functional load distribution. Huang et al. also emphasized the importance of multidisciplinary rehabilitation, including endodontic, periodontal, and prosthodontic procedures, to achieve favorable outcomes in complex anterior dental injuries [7].

The favorable one-year follow-up observed in the present case demonstrated that even severe iatrogenic injuries involving extensive root and bone destruction may be managed successfully through timely diagnosis, meticulous treatment planning, and coordinated interdisciplinary care [13]. This case further highlights the critical role of CBCT in surgical planning and reinforces the need for cautious operative techniques during mesiodens extraction to prevent irreversible complications.

## Conclusions

This case highlights the potential severity of iatrogenic complications associated with mesiodens extraction when adequate diagnostic evaluation and meticulous surgical planning are not performed. Improper surgical management results in extensive damage to the maxillary anterior teeth and supporting alveolar structures, compromising both function and esthetics. However, the affected dentition was successfully preserved through a carefully planned multidisciplinary approach involving endodontic therapy, surgical intervention, splinting, and prosthodontic rehabilitation. The favorable clinical and radiographic outcomes observed during the one-year follow-up emphasize the importance of conservative tooth-preserving strategies whenever feasible. Furthermore, this case highlights the critical role of CBCT in the preoperative assessment and surgical guidance of impacted supernumerary teeth. Accurate diagnosis, interdisciplinary collaboration, and long-term follow-up are essential to minimize complications and ensure predictable treatment outcomes for complex iatrogenic dental injuries.
